# Deletion of lysophosphatidylcholine acyltransferase 3 in myeloid cells worsens hepatic steatosis after a high-fat diet

**DOI:** 10.1194/jlr.RA120000737

**Published:** 2020-12-17

**Authors:** Thibaut Bourgeois, Antoine Jalil, Charles Thomas, Charlène Magnani, Naig Le Guern, Thomas Gautier, Jean-Paul Pais de Barros, Victoria Bergas, Hélène Choubley, Loïc Mazzeo, Louise Menegaut, Lorène Josiane Lebrun, Kévin Van Dongen, Marion Xolin, Tony Jourdan, Chloé Buch, Jérome Labbé, Philippe Saas, Laurent Lagrost, David Masson, Jacques Grober

**Affiliations:** 1 Univ. Bourgogne Franche-Comté, LNC UMR12131, Dijon, France; 2 INSERM, LNC UMR 1231, Dijon, France; 3 FCS Bourgogne Franche-Comté, LipSTIC LabEx, Dijon, France; 4 Lipidomic analytic plate-forme, Univ. Bourgogne Franche-Comté, Batiment B3, Bvd Maréchal de Lattre de Tassigny, Dijon, France; 5 AgroSup Dijon, Dijon, France; 6 Univ. Bourgogne Franche-Comté, INSERM, EFS BFC, UMR1098, Interactions Hôte Greffon-Tumeur/Ingénierie Cellulaire et Génique, LabEx LipSTIC, Besançon, France; 7 CHU Dijon, laboratoire de Biochimie, Dijon, France

**Keywords:** phospholipid, arachidonic acid, macrophages, atherosclerosis, steatosis, lysophosphatidylcholine acyltransferase 3 (LPCAT3), lipid metabolism, obesity, inflammation, insulin resistance, AA, arachidonic acid, LPCAT3, lysophosphatidylcholine acyltransferase 3, LPLAT, lyso-PL-acyltransferase, LPS, lipopolysaccharide, LXR, liver X receptor, PC, phosphatidylcholine, PL, phosphatidylinositol, pPE, plasmalogen, PS, phosphatidylserine

## Abstract

Recent studies have highlighted an important role for lysophosphatidylcholine acyltransferase 3 (LPCAT3) in controlling the PUFA composition of cell membranes in the liver and intestine. In these organs, LPCAT3 critically supports cell-membrane-associated processes such as lipid absorption or lipoprotein secretion. However, the role of LPCAT3 in macrophages remains controversial. Here, we investigated LPCAT3's role in macrophages both in vitro and in vivo in mice with atherosclerosis and obesity. To accomplish this, we used the *LysMCre* strategy to develop a mouse model with conditional *Lpcat3* deficiency in myeloid cells (*Lpcat3KO*^*Mac*^). We observed that partial *Lpcat3* deficiency (approximately 75% reduction) in macrophages alters the PUFA composition of all phospholipid (PL) subclasses, including phosphatidylinositols and phosphatidylserines. A reduced incorporation of C20 PUFAs (mainly arachidonic acid [AA]) into PLs was associated with a redistribution of these FAs toward other cellular lipids such as cholesteryl esters. *Lpcat3* deficiency had no obvious impact on macrophage inflammatory response or endoplasmic reticulum (ER) stress; however, *Lpcat3KO*^*Mac*^ macrophages exhibited a reduction in cholesterol efflux in vitro. In vivo, myeloid *Lpcat3* deficiency did not affect atherosclerosis development in LDL receptor deficient mouse (*Ldlr*^*−/−*^) mice. *Lpcat3KO*^*Mac*^ mice on a high-fat diet displayed a mild increase in hepatic steatosis associated with alterations in several liver metabolic pathways and in liver eicosanoid composition. We conclude that alterations in AA metabolism along with myeloid *Lpcat3* deficiency may secondarily affect AA homeostasis in the whole liver, leading to metabolic disorders and triglyceride accumulation.

Phospholipids (PLs) are continuously remodeled in a succession of deacylation and reacylation reactions called the Lands cycle (1). Turnover of fatty acids (FAs) at sn-2 position of PLs is mediated by the opposite actions of phospholipases A2 and lyso-PL-acyltransferases (LPLATs). Each LPLAT action is tissue- and substrate-dependent (2, 3). One of these enzymes, the lysophosphatidylcholine acyltransferase 3 (LPCAT3), is highly expressed in the liver and the intestine but also in macrophages (4, 5, 6). It has been shown that LPCAT3 promotes the insertion of polyunsaturated fatty acid (PUFAs), mainly arachidonic acid (AA), at the sn-2 position of cellular PLs (5, 6). LPCAT3 expression is dynamically regulated by nuclear receptors such as liver X receptors (LXRs) and peroxisome proliferator-activated receptors (PPARs) (4, 7, 8). Deletion of LPCAT3 decreases the abundance of PUFAs within PLs leading to alterations of the physicochemical properties of cell membranes. In mouse models, *Lpcat3* deletion is associated with alterations of several membrane-associated processes such as lipoprotein assembly and secretion by hepatocytes (5, 6), dietary lipid absorption (5, 6, 9), and SREBP1c cleavage (10). An acute inhibition of *Lpcat3* was shown to promote ER stress and inflammation in the liver (8), whereas it was not observed in *Lpcat3*^*−/−*^ mice. In the mouse, constitutive *Lpcat3* deficiency is lethal few days after birth probably due to the malabsorption of dietary lipids (5, 9, 11). While these studies focused mainly on the liver and intestine, the impact of LPCAT3 on macrophage functions is more controversial. While acute *Lpcat3* inhibition in murine peritoneal macrophages was shown to potentiate the inflammatory response to lipopolysaccharides (LPSs) (4), siRNA-mediated knockdown of *LPCAT3* in human primary macrophages did not affect the overall inflammatory response but decreased eicosanoid secretion, probably due to a depletion of AA in membrane phospholipids (8). Phenotypic analysis of *Lpcat3*-deficient mouse macrophages also revealed some discrepancies between the studies. Indeed, a complete *Lpcat3* deficiency in fetal liver-derived macrophages did not affect the inflammatory response or ER stress but was shown to affect eicosanoid secretion and cholesterol homeostasis (12). The latter point was associated with an inhibition of cholesterol efflux pathways. In contrast, in bone-marrow-derived macrophages (BMDMs) from *Lpcat3*-^Flox^/LysM-Cre mice presenting with a 20% residual *Lpcat3* activity, it was observed an increase in *Il1b* mRNA levels and IL-6 and TNF-α secretion following LPS stimulation while some markers of ER stress were significantly decreased (13). Finally, in a study using the same *Lpcat3*-^Flox^/LysM-Cre mouse model, *Lpcat3* deficiency did not affect *Il1b* mRNA levels and significantly reduced *Cox2* mRNA levels following LPS stimulation (14). To clarify the function of LPCAT3 in macrophages in vitro and in vivo, we generated a mouse model with a conditional *Lpcat3* deficiency in myeloid cells (*Lpcat3KO*^*Mac*^ mice) presenting with an 80% reduction of *Lpcat3* expression in macrophages. Moreover, we investigated the impact of macrophage *Lpcat3* deficiency in mouse models of atherosclerosis and obesity/hepatic steatosis. Here, we showed that a partial deletion of *Lpcat3* in macrophages significantly affects the FA composition of all phospholipid subclasses as well as the distribution of AA within the cellular lipids, but did not alter the inflammatory response in vivo or in vitro. While *Lpcat3* macrophage deficiency did not affect atherosclerosis development, *Lpcat3KO*^*Mac*^ mice presented with a mild increase in hepatic steatosis under a high-fat diet (HFD) that was associated with alterations of several metabolic pathways and liver eicosanoid composition.

## Materials and methods

### Generation of myeloid-cell-specific *Lpcat3*-deficient mice

*Lpcat3*^*fl/fl*^ mice (12) were crossed with *LysM*^*Cre/+*^ transgenic mice to obtain *Lpcat3*^*fl/fl*^*/LysM*^*Cre/+*^ (*Lpcat3KO*^*Mac*^) and *Lpcat3*^*fl/fl*^*/LysM*^*+/+*^ littermate (WT). We used male and female mice on a C57BL/6N background. All animal procedures were performed in accordance with institutional guidelines and approved by the University of Burgundy's Ethics Committee on the Use of Laboratory Animals (protocol number 8381). At the age of 8–12 weeks old, mice were either maintained on a chow diet (CD) (A3, Safe) or fed an HFD for 16 weeks (60% fat diet, D12492, Ssniff) or a western diet for 12 weeks (TD88137, Harlan Teklad).

### Bone marrow transplantation

Eight-week-old *Ldlr*^*−/−*^ mice were lethally irradiated with 1,000 rads (10 Gy) before transplantation. Recipient mice were injected with about 2 × 10^6^ bone-marrow-derived monocytes through the tail vein. Recipient *Ldlr*^*−/−*^ mice were given acidified water (pH 4.5) containing enrofloxacin 0.25% for 2 weeks after transplantation.

### Atherosclerosis study

All experiments were performed according to already published protocols (12).

### In vivo metabolic tests

Glucose (2 g/kg, oral administration), pyruvate (2 g/kg, i.p.), and insulin (0.4–0.8 U/kg, i.p.) tolerance tests were performed after a 6-h fast (OGTT, ITT) or overnight fast (PTT). Glycemia is measured with glucometer (Accu-Chek Performa).

### LPS treatment

LPSs from *Escherichia coli* 055:B5 (L2880, Sigma) were solubilized in saline 0.9% and injected i.p. in mice at 1 mg/kg. Treatment of culture cells was performed at a concentration of 100 ng/ml.

### BMDM preparation

Anesthetized mice were euthanized by cervical dislocation. Bone marrow in femur and tibia was flushed, and 300,000 bone marrow cells were implanted in 12-well plates. Cells were treated during 5–7 days with human M-CSF (130-096-492, Miltenyi) until full macrophage differentiation.

### Isolation of Kupffer cells and primary hepatocytes

Mice were anesthetized, and a two-step collagenase perfusion of the liver was performed as previously described (15). Digested livers were centrifugated (30 *g*, 3 min, 4°C). Briefly, cell pellets containing primary hepatocytes were washed and divided for mRNA analysis and metabolic activity assay (Substrate palmitate-BSA FAO Seahorse XF, Agilent) on SeaHorse XFe96 Analyzer (Agilent). Primary hepatocytes were plated in FAO Assay Buffer according to manufacturer protocol (Krebs Henseleit Buffer: 111 mM NaCl, 4.7 mM KCl, 1.25 mM CaCl_2_, 2 mM MgSO_4_, 1.2 mM NaH_2_PO_4_, 2.5 mM glucose, 0.5 mM carnitine, and 5 mM HEPES) and were treated with control BSA or 0.17 mM palmitate:BSA (6:1 ratio) prior to the XF assay being initiated (t = 0). Oxygen consumption rate was measured in basal conditions and after injections of oligomycin, FCCP, and Rotenone/Antimycin A. Supernatants containing Kupffer cells were used for cell sorting using MACS (Anti-F4/80 MicroBeads UltraPure, mouse, Miltenyi) and analyzed by RNA sequencing (Genewiz, Germany, GEO number: GSE146004).

### Lipidomic analyses

Phosphatidylcholine, phosphatidylethanolamine, cholesterol, and FA analyses were performed at the lipidomic platform of Dijon (France) according to protocols already published (12). Phosphatidylinositols and phosphatidylserines were analyzed by LCMS/MS using the same chromatographic conditions as previously described (12). Acquisition was performed on an Agilent 6460 QqQ mass spectrometer in negative selected reaction monitoring ion mode (source temperature 325°C, nebulizer gas flow rate 10 L/min, sheath gas flow 11 L/min, temperature 300°C, capillary 3500 V, nozzle 1000 V). Fragmentor was set up at 172 V and 150 V for phosphatidylinositols and phosphatidylserines, respectively. Collision energy was set up at 50 V and 19 V for phosphatidylinositols and phosphatidylserines, respectively. Each glycerophospholipid was semiquantitated by calculating their response ratio with regard to their respective internal standard.

### Biochemical analyses

Plasma insulin and GLP-1 were measured with ELISA kit (STELLUX® Chemiluminescent Rodent Insulin ELISA ALPCO and EZGLP1T-36K, Millipore, respectively). Triglycerides (TGs), cholesterol, and free fatty acid (FFA) concentrations were measured by enzymatic methods (Triglycerides FS, Cholesterol FS, NEFA FS, Diasys). Cytokines were measured with a Milliplex MAP 5-Plex Kit using mouse cytokine/chemokine magnetic bead panel (MCYTOMAG-70K, Millipore), according to the manufacturer's protocol, and using a LuminexR apparatus (Bio-Plex 200, Bio-Rad).

### Cholesterol efflux

Cholesterol efflux experiments were performed according to published protocol (12).

### Real-time quantitative PCR

Tissues were immediately frozen in liquid nitrogen and stored at –80°C. Cell culture lysates were directly stored at –80°C. Total RNA was isolated using RNeasy Mini Kit (74106, Qiagen) and quantified by spectrophotometer (Nanodrop 1000, Thermo Scientific). In total, 100–1000 ng RNA was reverse transcribed using High-Capacity cDNA Reverse Transcription Kit (Multiscribe® reverse transcriptase, 4368813, Applied Biosystems), and quantitative PCR were performed using StepOnePlus (Real-Time PCR System, Applied Biosystems) and SYBRGreen® (4367659, Applied Biosystems) technologies. The mRNA levels were normalized with housekeeping gene *Rplp0* and expressed as relative expression using the 2^*−*ΔΔCt^ method.

### Statistical analyses

Data are presented as mean ± SEM. Statistical analyses were performed using GraphPad Prism. To decide whether to use parametric or nonparametric statistics, the normality of distributions was assessed with the Shapiro-Wilk test (under n = 7, distributions were considered to be nonnormal). Statistical significance of differences between two groups was evaluated with the Mann-Whitney U test or the Student's *t*-test. A value of *P* < 0.05 was considered statistically significant (NS, not significant; ∗*P* < 0.05, ∗∗*P* < 0.01, and ∗∗∗*P* < 0.001).

## Results

### Lipidomic characterization of *Lpcat3KO*^*Mac*^ cells

**Fig. 1:**
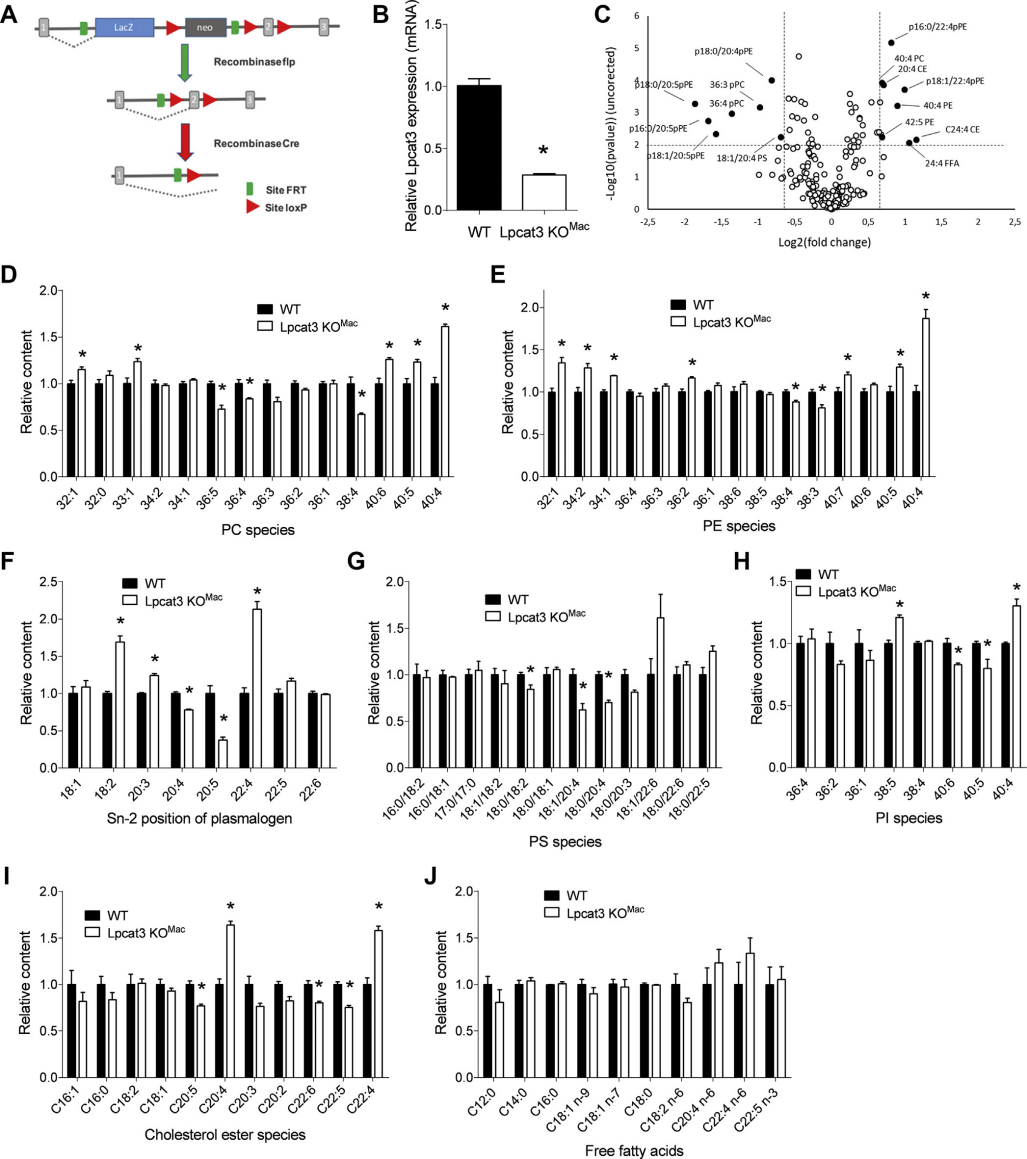
Generation of *Lpcat3KO*^*Mac*^ mice and lipidomic characterization of Lpcat3-deficient macrophages. A: *Lpcat3* targeting vector. A gene-trap LacZ cassette is located downstream of exon 2 of the *Lpcat3* gene. B: Relative *Lpcat3* mRNA levels in macrophages derived from WT and *Lpcat3KO*^*Mac*^ mice (four independent mice in each group). Data are expressed as mean ± SEM (∗*P* < 0.05 vs. WT Mann-Whitney test). C: Changes of lipidomic profile of *Lpcat3KO*^*Mac*^ versus WT macrophages (four independent mice in each group, *P* value at 0.01 and ±0.6 log-fold changes as cut off). D–J: Fatty acid composition of phosphatidylcholines (D), phosphatidylethanolamines (E), plasmalogens (F), phosphatidylserines (G), phosphatidylinositols (H), cholesterol esters (I), and free fatty acids (J) of WT and *Lpcat3KO*^*Mac*^ macrophages (n = 4 in each group). Data are expressed as mean + SEM (∗*P* < 0.05 vs. WT Mann-Whitney test).

We generated a transgenic model of mice deficient for *Lpcat3* in myeloid cells (*Lpcat3KO*^*Mac*^ mice) by crossing *Lpcat3-Flox* mice with *LysMCre* transgenic mice (Fig. 1A). In isolated BMDMs, *Lpcat3* mRNA levels were reduced by approximately 75% in *Lpcat3KO*^*Mac*^ cells as compared with controls (*Lpcat3-Flox*) (Fig. 1B). To analyze the impact of *Lpcat3* deficiency on the lipid composition of BMDMs, we conducted a targeted lipidomic analysis of 244 molecules including major phospholipid subclasses (phosphatidylcholines [PCs], phosphatidylserines [PSs], phosphatidylethanolamines [PEs], phosphatidylinositols [PIs], plasmalogens [pPEs], total FAs, FFAs, and cholesteryl esters) (supplemental Fig. S1). By using a *P* value of 0.01 and ±0.6 log-fold changes as a cutoff, we found that the levels of 14 molecules were significantly altered in *Lpcat3KO*^*Mac*^ cells (Fig. 1C). As expected, C20:4 n-6 and C20:5 n-3 containing phospholipids were among the molecules that were significantly reduced in *Lpcat3KO*^*Mac*^ macrophages, while C22:4 containing phospholipids and C20:4 cholesteryl esters were increased. Analysis of phospholipid subclasses revealed that in addition to PCs, Pes, and pPEs (Fig. 1D–F) that were previously characterized as LPCAT3 substrates, the composition of PSs (Fig. 1G) and PIs (Fig. 1H) was also significantly affected by *Lpcat3* deficiency. C20:4 depletion in phospholipids was associated with a redistribution of AA toward cholesteryl esters (Fig. 1I), as previously observed (12). However, as compared with total *Lpcat3* deficiency (12), changes in FA composition of PLs were less pronounced in *Lpcat3KO*^*Mac*^ cells, and there was no significant increase in non-esterified FA levels (Fig. 1J) including AA and C22:4 n-6 (i.e., a direct elongation product of AA) as it was the case in *Lpcat3*^*−/−*^ macrophages (12). Nevertheless, our data demonstrate that even a partial *Lpcat3* deficiency markedly affects AA homeostasis and distribution in macrophages.

### Inflammatory response and ER stress of *Lpcat3KO*^*Mac*^ cells

**Fig. 2:**
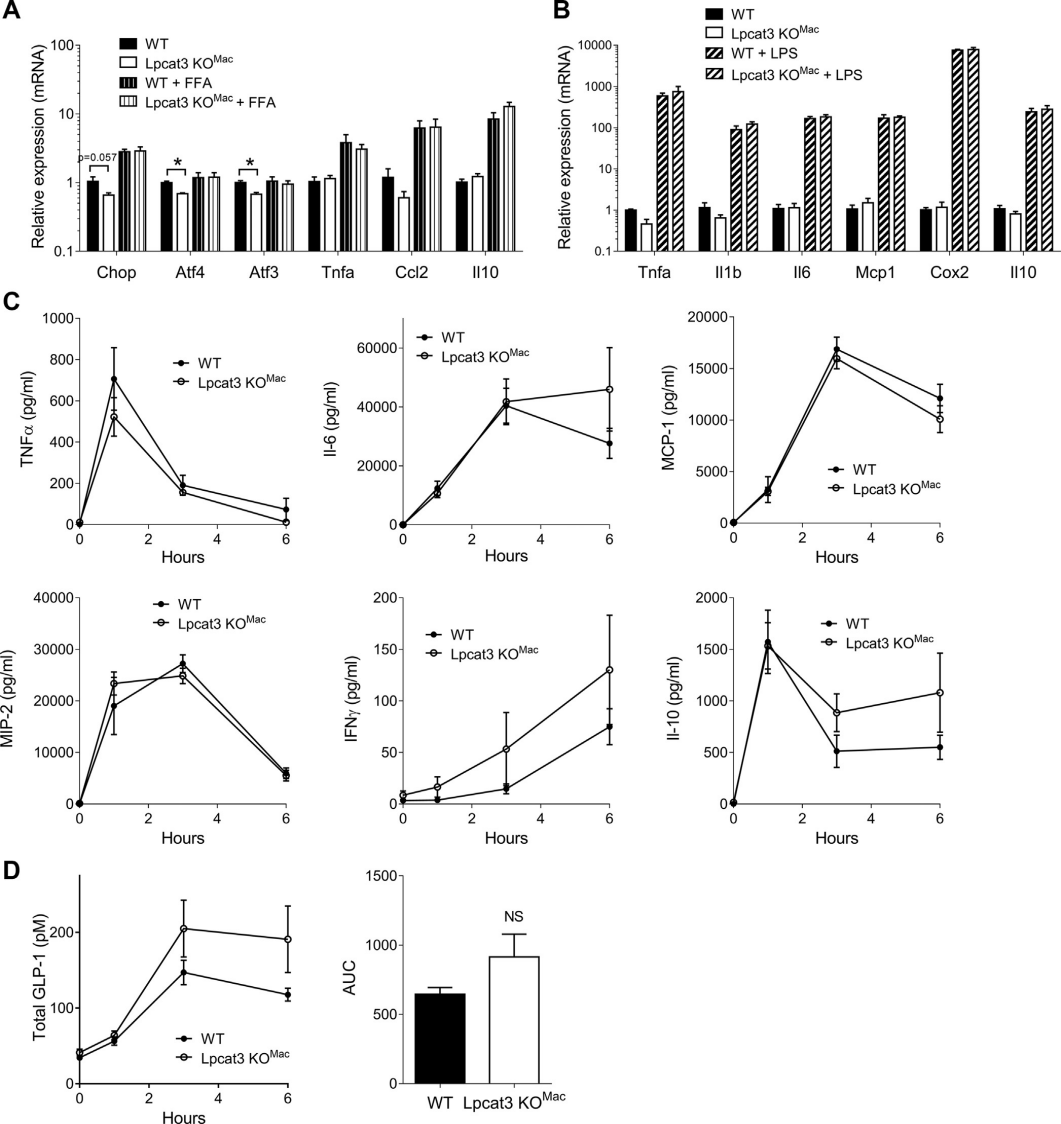
ER stress and inflammatory response in *Lpcat3KO*^*Mac*^ mice. A: Relative mRNA levels of ER stress markers or inflammatory genes in macrophages from WT and *Lpcat3KO*^*Mac*^ mice and treated or not with 200 μM free fatty acids (oleate and palmitate) (n = 4 independent mice in each group). B: Relative expression of pro- or anti-inflammatory genes in macrophages from WT and *Lpcat3KO*^*Mac*^ mice treated or not with 100 ng/ml LPS (n = 4 independent mice in each group) Data are expressed as mean + SEM. (∗*P* < 0.05 vs. WT Mann-Whitney test by treatment condition). C, D: Plasma concentration of cytokines (C) and total GLP-1 (D) in WT and *Lpcat3KO*^*Mac*^ mice treated with 1 mg/kg LPS (n = 6 and 5, respectively). Data are expressed mean ± SEM (∗*P* < 0.05 vs. WT Mann-Whitney test for AUC).

It has been suggested that LPCAT3-dependent membrane remodeling may affect ER stress (8) and inflammation (13). We evaluated the impact of *Lpcat3* deficiency on these two biological processes under both basal and stimulated conditions. Interestingly, *Lpcat3KO*^*Mac*^ macrophages presented a slight but significant decrease in the expression of ER stress markers at basal state; however, after loading of the macrophages with saturated FFAs to induce ER stress, there were no differences between the two genotypes (Fig. 2A). No differences were observed between WT and *Lpcat3KO*^*Mac*^ cells regarding inflammatory gene expression under either control, or FFA loading, or LPS-stimulated conditions (Fig. 2A, B). To evaluate the inflammatory response in vivo, WT and *Lpcat3KO*^*Mac*^ mice were challenged with LPS injection and plasma cytokines were evaluated up to 6 h after LPS injection. In accordance with our in vitro observations, we did not find any differences in cytokine secretion (Fig. 2C). Furthermore, secretion of GLP-1, which is produced by enteroendocrine cells in response to LPS, was not significantly increased in *Lpcat3KO*^*Mac*^ mice treated with LPS (Fig. 2D). Our results suggest therefore that while ER stress could be slightly reduced in *Lpcat3KO*^*Mac*^ macrophages, there is no obvious impact on acute inflammatory response in vitro and in vivo.

### Partial *Lpcat3* deficiency restricted to myeloid cells does not impact atherosclerosis development

**Fig. 3:**
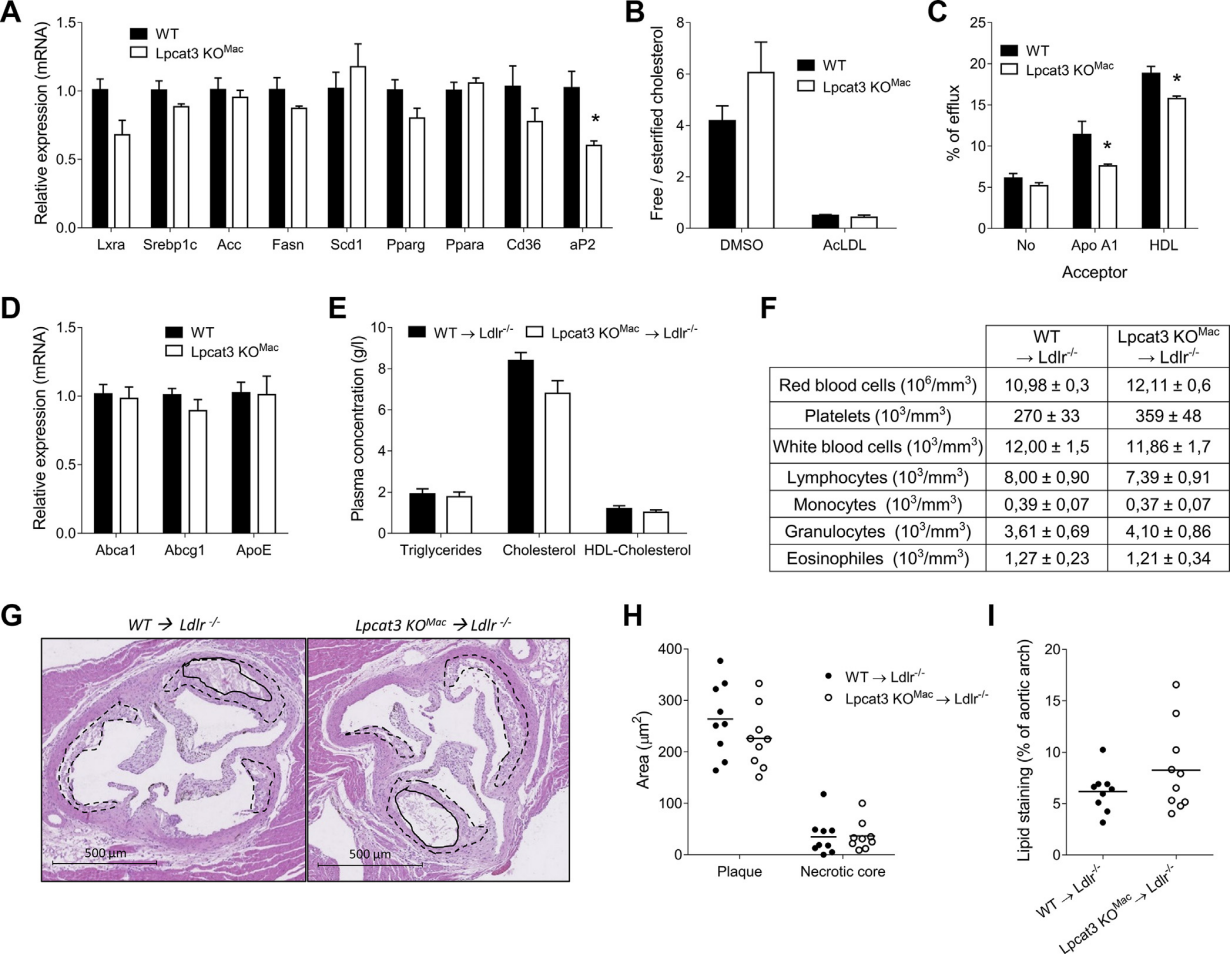
Partial *Lpcat3* deficiency restricted to myeloid cells does not induce atherosclerosis. A: Relative mRNA levels of genes involved in lipid metabolism (n = 4 vs. 4). B: Ratio of free to esterified cholesterol in WT and *Lpcat3KO*^*Mac*^ mouse-derived macrophages treated or not with acetylated LDL (n = 4 vs. 4 independent mice in each group). C: Cholesterol efflux with lipid-free ApoA-I or HDL was assessed in [3H] cholesterol-acetylated LDL loaded macrophages, n = 3 independent experiments. D: Relative mRNA levels of *Abca1*, *Abcg1* and *ApoE* (n = 9 in each group) in WT and *Lpcat3KO*^*Mac*^ mouse-derived macrophages. E: Plasma lipid parameters from recipient Ldlr^*−*/*−*^ mice transplanted with WT and *Lpcat3KO*^*Mac*^ BMDM cells at 14 weeks of Western-type diet (n = 9 in each group). F: Blood cell counts from recipient Ldlr^*−*/*−*^ mice transplanted with WT and *Lpcat3KO*^*Mac*^ bone marrow cells (n = 9 in each group). G, H: HE staining of aortic valves from *Ldlr*^*−/−*^ recipient mice fed with a Western-type diet for 14 weeks. G: Dotted line, atheroma plaque; continuous line, necrotic core area. H: Analysis of plaque and necrotic core size. I: % of Oil Red O stained area in aortic arches of Ldlr^*−*/*−*^ recipient mice fed a 12 week WTD. Values are expressed mean + SEM (∗*P* < 0.05, ∗∗*P* < 0.01 vs. WT Mann-Whitney test).

Next, we wanted to address the impact of *Lpcat3* deficiency on macrophage lipid homeostasis and atherosclerosis development. Targeted transcriptomic analysis of *Lpcat3KO*^*Mac*^ BMDMs did not revealed major alterations in genes involved in FA and cholesterol metabolism at the exception of *Ap2* (*Fabp4*) that was significantly decreased in *Lpcat3KO*^*Mac*^ macrophages (Fig. 3A). Interestingly, there was a significant decrease in cholesterol efflux toward ApoA1 and HDL in *Lpcat3KO*^*Mac*^ macrophages as compared with WT cells (Fig. 3C). Since *Abca1* and *Abcg1* mRNA levels were similar in WT and *Lpcat3KO*^*Mac*^ macrophages (Fig. 3D), these data suggest that *Lpcat3* deficiency may affect cholesterol efflux by altering cell membrane lipid composition. To evaluate the impact of *Lpcat3* macrophage deficiency on atherosclerosis development, *Ldlr*^*−/−*^ mice were lethally irradiated and then transplanted with hematopoietic cells harvested from WT or *Lpcat3KO*^*Mac*^ bone marrows. After 4 weeks of recovery, the mice were fed with a Western-type diet (WTD) for 14 weeks. There was no difference in weight gain during the diet (data not shown), and the two groups of mice displayed similar plasma lipid concentrations (Fig. 3E). There was no difference in peripheral blood leukocyte counts, including monocytes, after 14 weeks of WTD (Fig. 3F). Analysis of atherosclerotic lesions after 14 weeks revealed similar lesion sizes in aortic roots in mice reconstituted with *Lpcat3KO*^*Mac*^ or WT bone marrow cells. There was also no difference in the necrotic core area (Fig. 3G, H). No difference was observed regarding atherosclerotic lesions in aortic arches (Fig. 3I).

### Weight gain is not affected by a myeloid *Lpcat3* deficiency under standard diet

**Fig. 4:**
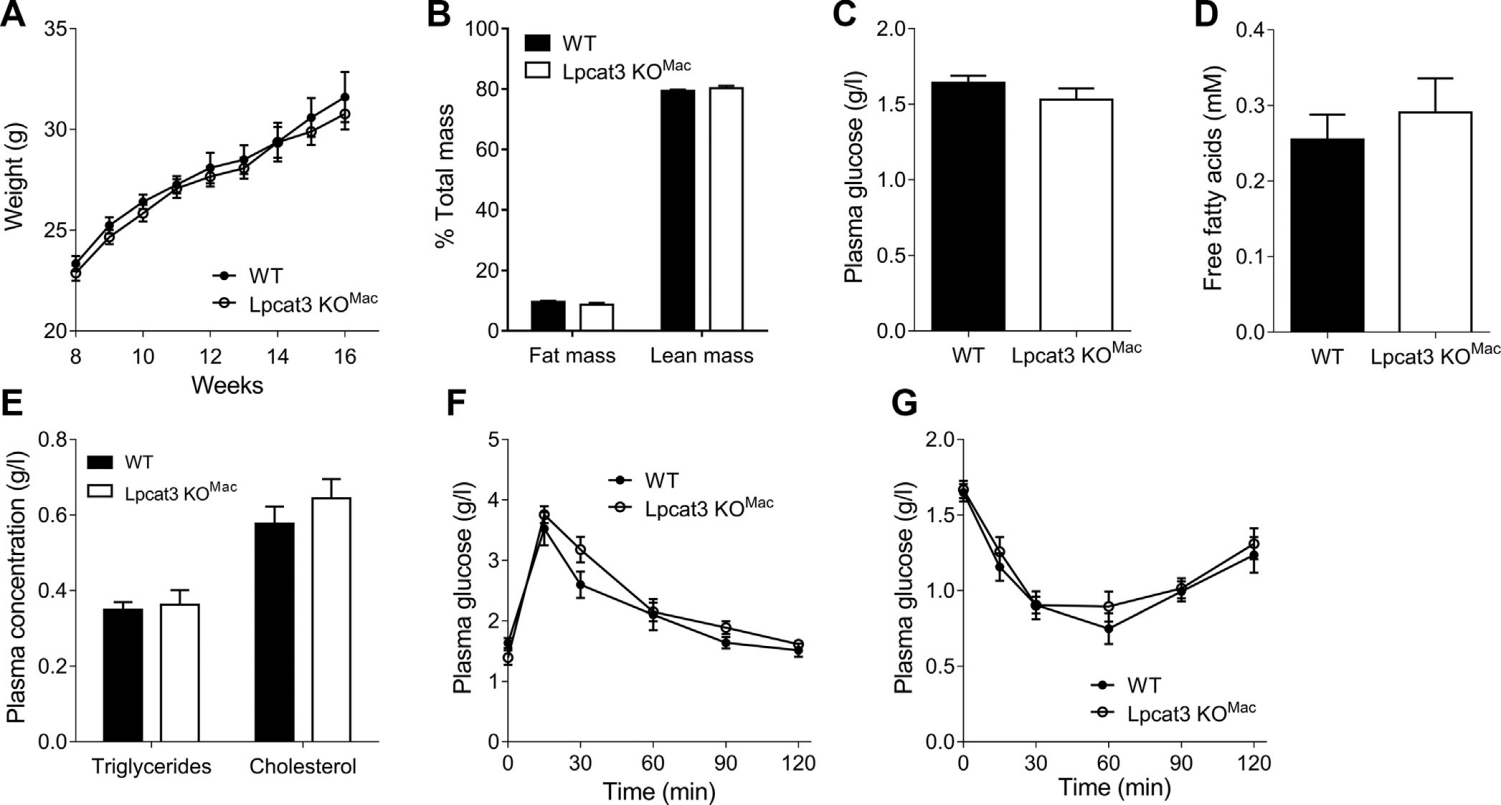
*Lpcat3KO*^*Mac*^ mice grow normally under a chow diet. A: Weight gain of mature WT and *Lpcat3KO*^*Mac*^ mice fed a chow diet (n = 7 vs. 13, respectively). B: Fat mass and lean mass of WT and *Lpcat3KO*^*Mac*^ mice after 8 weeks of chow diet (n = 7 vs. 13, respectively). C–E: Plasma glucose (C), free fatty acids (D) and triglycerides, and cholesterol (E) were assessed in 6 h fasting WT and *Lpcat3KO*^*Mac*^ mice (n = 6 in each group). F, G: Oral glucose tolerance test (F) and insulin tolerance test (G) of 6 h fasting WT and *Lpcat3KO*^*Mac*^ mice after 8 weeks of chow diet (n = 6 in each group). Data are expressed mean ± SEM (∗*P* < 0.05 vs. WT Mann-Whitney test or Mann-Whitney test for AUC).

Obesity is another chronic inflammatory disease in which macrophages are responsible for progression of chronic inflammation and insulin resistance (16). We investigated the impact of macrophage-specific *Lpcat3* deficiency on progression of obesity. First, under a CD, we observed that *Lpcat3KO*^*Mac*^ and WT mice grow at a similar rate (Fig. 4A). No changes in fat or lean mass were observed (Fig. 4B). No differences were observed in blood fasting glucose (Fig. 4C), FFAs (Fig. 4D) or TGs, and cholesterol (Fig. 4E) between *Lpcat3KO*^*Mac*^ and WT mice. Glucose metabolism was assessed through in vivo oral glucose tolerance test (OGTT) and insulin tolerance test (ITT). As shown in Figure 4F, no differences were observed with these two different tests. According to these results, *Lpcat3KO*^*Mac*^ mice fed a CD thrive normally without obvious alterations in glucose or lipid metabolism.

### *Lpcat3KO*^*Mac*^ mice develop hepatic steatosis under high-fat diet

**Fig. 5:**
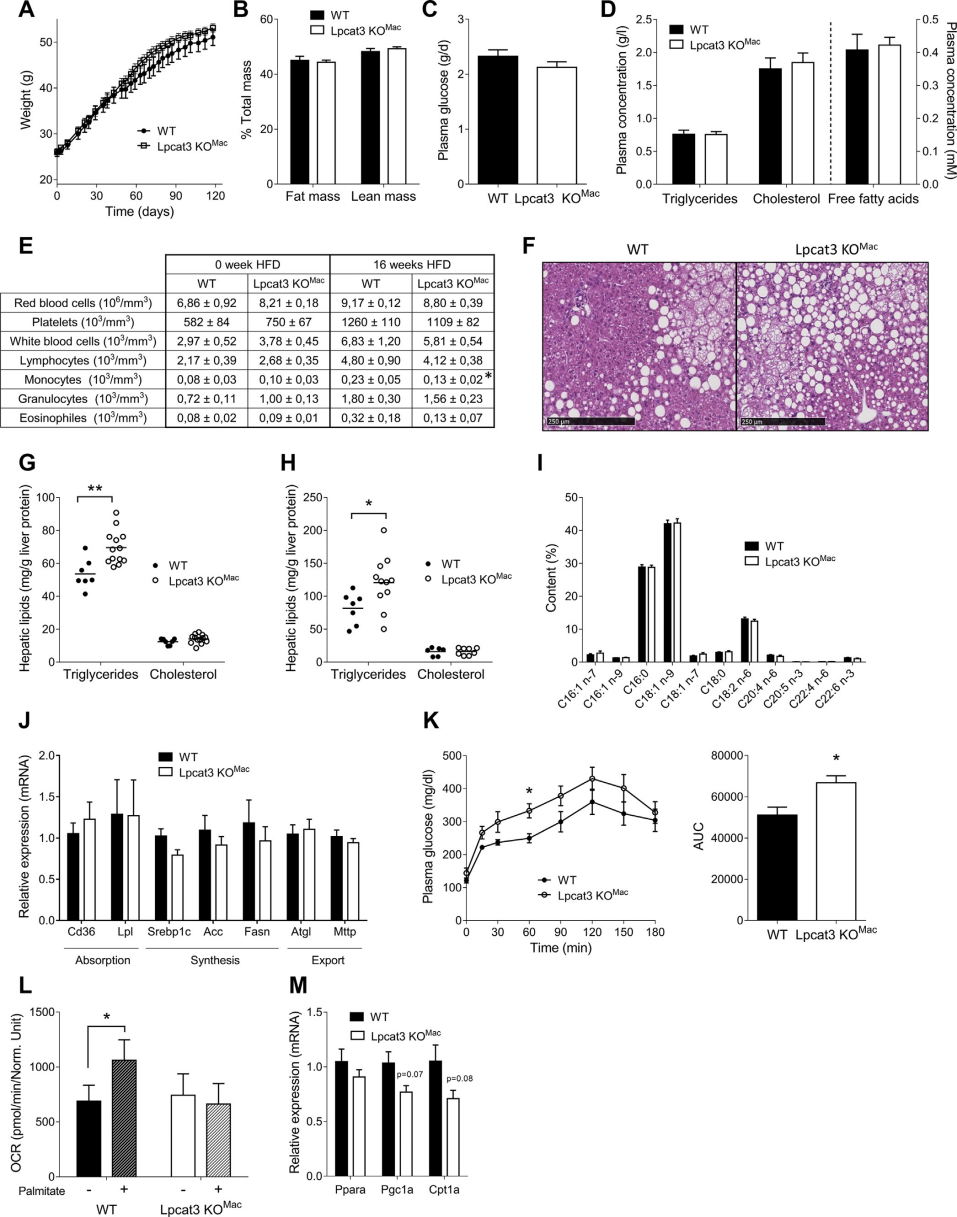
*Lpcat3KO*^*Mac*^ mice suffer from hepatic steatosis when fed a high-fat diet. A: Weight gain of mature WT and *Lpcat3KO*^*Mac*^ mice fed a high-fat diet (n = 7 and 13, respectively). B: Fat mass and lean mass of WT and *Lpcat3KO*^*Mac*^ mice after 16 weeks of high-fat diet (n = 7 and 13, respectively). Plasma glucose (C), free fatty acids and triglycerides, and cholesterol (D) were assessed in 6 h fasting WT and *Lpcat3KO*^*Mac*^ mice at the end of the diet (n = 7 and 13, respectively). E: Blood cell counts at the beginning and the end of HFD (n = 7 and 13, respectively). F: HE staining of liver of WT and *Lpcat3KO*^*Mac*^ mice after 16 weeks of high-fat diet. G, H: Measurements of lipid content in the liver of males (G, n = 7 vs. 13) and females (H, n = 7 vs. 11). I: Total fatty acid content in the liver of WT and *Lpcat3KO*^*Mac*^ mice (n = 7 vs. 13). J: Relative mRNA levels of genes involved in liver lipid metabolism pathways (n = 7 vs. 13). K: Pyruvate tolerance test of overnight fasting WT and *Lpcat3KO*^*Mac*^ mice and area under cover of this PTT. L: Oxygen consumption rate of primary hepatocytes of WT and *Lpcat3KO*^*Mac*^ mice, treated or not with palmitate. M: Liver relative mRNA levels of genes involved in fatty acid oxidation at the end of the HFD (n = 7 vs. 13). Data are expressed mean ± SEM (∗*P* < 0.05 vs. WT Mann-Whitney test).

The impact of an HFD on obesity progression was then explored in *Lpcat3KO*^*Mac*^ mice. While the % of fat mass increased dramatically after 4 months of HFD, body mass and body composition (Fig. 5A, B) as well as food intake (supplemental Fig. S2A) were not different between the two genotypes (Fig. 5B). *Lpcat3* deficiency did not affect the blood parameters measured. Indeed, fasting plasma glucose (Fig. 5C), TGs, cholesterol, and FFA concentrations (Fig. 5D) were unchanged compared with WT mice. Interestingly, number of circulating monocytes was reduced in *Lpcat3KO*^*Mac*^ mice at the end of the diet, whereas it was not different at the beginning (Fig. 5E). As observed under a CD, no differences were observed with OGTT or ITT (supplemental Fig. S2B–D). Fat storage was then assessed in the adipose tissue and liver. While no difference was observed in the adipose tissue mass, adipocyte size, or expression of genes involved in adipocyte metabolism (supplemental Fig. S3), livers of *Lpcat3KO*^*Mac*^ mice displayed significant alterations. Indeed, histological sections of livers from *Lpcat3KO*^*Mac*^ mice showed higher macrovesicular steatosis as compared with WT mice (Fig. 5F). Liver lipid content was measured, and we observed a mild but significant increase of TGs in both males and females *Lpcat3KO*^*Mac*^ mice; in contrast, cholesterol content remained unchanged (Fig. 5G, H). The liver FA profile was similar in the two groups of mice, with palmitic acid (C16:0) and oleic acid (C18:1) representing the majority of the FAs (Fig. 5I). No changes in the expression of genes involved in lipid absorption, synthesis, or export were observed in the liver (Fig. 5J), whereas a pyruvate tolerance test revealed an increase in the conversion of pyruvate to glucose in *Lpcat3KO*^*Mac*^ mice compared with WT mice (Fig. 5K). FA oxidation was investigated in primary hepatocytes isolated after 16 week HFD (Fig. 5L). An increase of oxygen consumption rate (OCR) was observed in hepatocytes from WT mice following palmitate addition, whereas no changes were observed in hepatocytes from *Lpcat3KO*^*Mac*^ mice. Genes involved in the FA oxidation pathway were not significantly reduced in *Lpcat3KO*^*Mac*^ hepatocytes despite a strong trend (Fig. 5M). In conclusion, under an HFD, *Lpcat3KO*^*Mac*^ mice suffer from hepatic steatosis that seemed associated with decreased FA oxidation and increased gluconeogenesis.

### Lipidomic and transcriptomic analyses reveal significant alterations of AA metabolism in the liver from *Lpcat3KO*^*Mac*^ mice

**Fig. 6:**
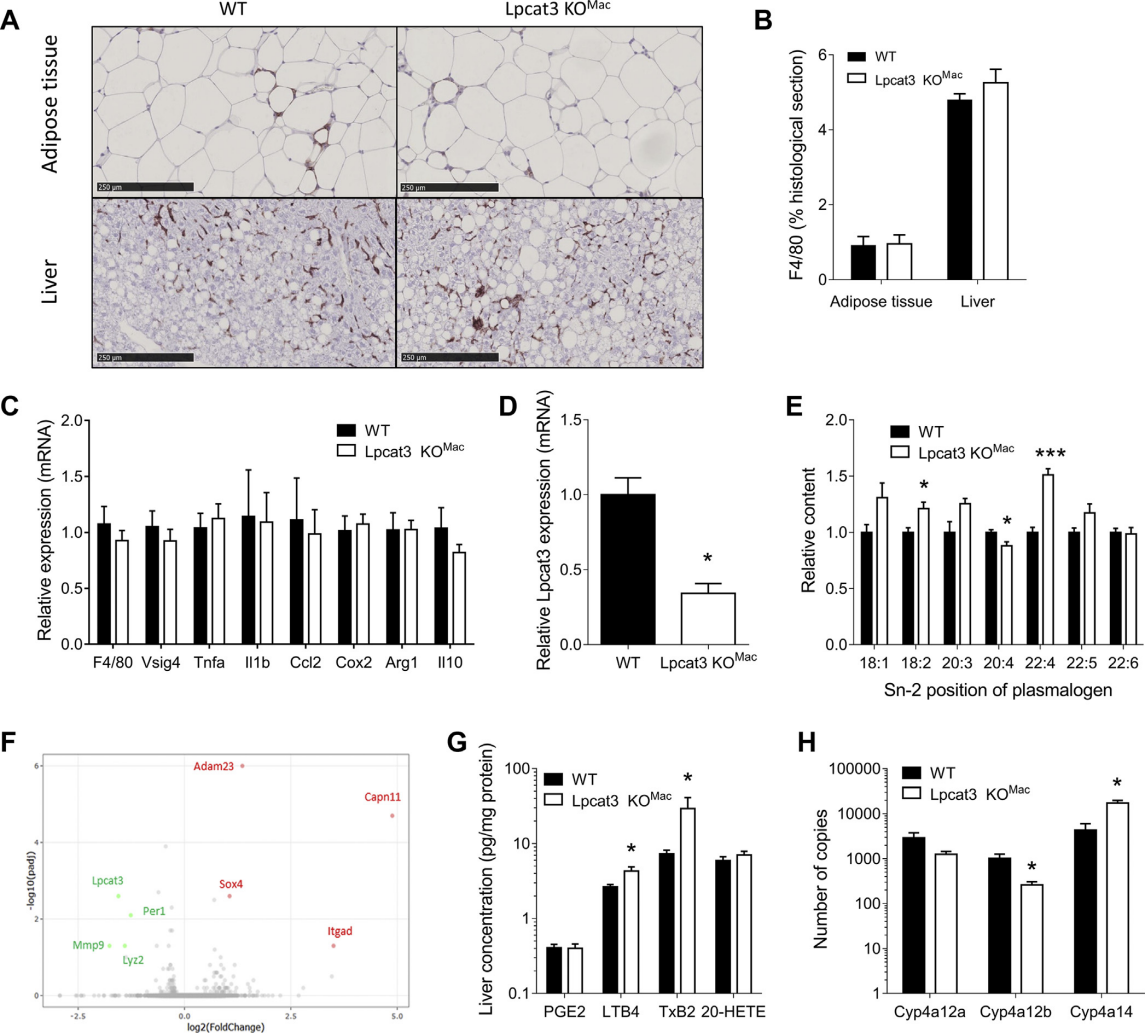
Mechanisms involved in hepatic steatosis progression in *Lpcat3KO*^*Mac*^ mice when fed a high-fat diet for 16 weeks. A, B: F4/80 staining in histological sections of adipose tissue and liver of Ctrl and *Lpcat3 KO*^*Mac*^ (n = 7 vs. 13, respectively). C: Liver mRNA levels of inflammatory genes of wild-type and *Lpcat3KO*^*Mac*^ mice (n = 7 vs. 13, respectively). D: Relative *Lpcat3* mRNA levels of in isolated Kupffer cells (n = 3 in each group). E: Relative content of fatty acid at the sn-2 position of pPE from isolated Kupffer cells after HFD. Data are expressed as a % of total pPE and are normalized as 1 in the WT group (n = 3 in each group). F: Volcano plot of differentially expressed genes in Kupffer cells at the end of the diet (n = 3 in each group). G: Eicosanoid content in the liver of WT and *Lpcat3KO*^*Mac*^ mice (n = 7 vs. 13, respectively). H: Relative mRNA levels of genes involved in Cytochrome P450 pathway in the whole liver (n = 4 in each group). Data are expressed as mean + SEM (∗*P* < 0.05 vs. WT Mann-Whitney test).

Histological sections stained with F4/80 antibodies were performed on the adipose tissue and liver after 4 months of HFD (Fig. 6A). There was no difference in macrophage infiltration in the adipose tissue and in the liver of *Lpcat3KO*^*Mac*^ mice as compared with WT mice (Fig. 6B). Furthermore, analysis of the sections did not reveal signs of liver inflammation and expressions of inflammatory genes were similar in the liver of *Lpcat3KO*^*Mac*^ mice fed an HFD as compared with control mice (Fig. 6C). Liver F4/80-positive cells were isolated by magnetic cell sorting (MACS). As expected, myeloid cells from *Lpcat3KO*^*Mac*^ mice displayed reduced *Lpcat3* mRNA levels (Fig. 6D). Lipidomic analysis confirmed the phenotype with a decrease of C20:4 n-6 and an increase of C18:2 n-6 and C22:4 n-6 at the sn-2 position of plasmalogens (Fig. 6E). The transcriptome of liver F4/80 positive cells was investigated by a global RNAseq approach. Surprisingly, a very low number of genes were found to be differentially expressed between the two genotypes, among them *Lpcat3* and *Lyz2* as expected. Notably, there was no difference in inflammatory genes as assessed by a PCR approach (Fig. 6F). Since F4/80 positive cells displayed an alteration in their AA composition, we measured the concentration of AA-derived mediators in the whole liver. Interestingly, eicosanoid profile was altered in the liver of *Lpcat3KO*^*Mac*^ mice with significant increases in leukotriene B4 (LTB4) and thromboxane B2 (TxB2) concentrations (Fig. 6G). While there were no changes in the expression of enzymes involved in leukotriene or thromboxane pathways in the Kupffer cells from *Lpcat3KO*^*Mac*^ mice, analysis of the whole liver tissue revealed significant alterations of CYP450 enzymes involved in AA metabolism (17) with a significant decrease of *Cyp4a12b* and increase of *Cyp4a14* mRNA levels (Fig. 6H). While these enzymes are not directly involved in eicosanoid synthesis, these data come in further support of an altered AA homeostasis in the liver of *Lpcat3KO*^*Mac*^ mice.

## Discussion

Recent studies have highlighted the major role of LPCAT3 in controlling the PUFA composition of cell membranes in the liver and intestine with dramatic consequences on cell-membrane-associated processes such as lipid absorption, lipoprotein secretion, and SREBP cleavage (5, 6, 11). In contrast, the role of LPCAT3 in myeloid cells and macrophages remains unclear (4, 8, 12, 13). In the present study, we developed a mouse model with conditional *Lpcat3* deficiency in myeloid cells by using the *LysMCre* strategy. Our aim was to investigate the function of LPCAT3 in macrophages in vitro but also in vivo in experimental models of atherosclerosis and obesity. Indeed, chronic low-grade inflammation mediated in part by macrophages and myeloid cells is known to play a key role in these two cardiometabolic diseases (18, 19).

As it is often the case with similar conditional models (20), crossing the *Lpcat3*^*flox/flox*^ mice with the *LysMcre* mice led to an incomplete inactivation of the *Lpcat3* gene in myeloid cells with a residual approximately 25% expression that was observed either in vitro or in vivo. Thus, our *Lpcat3KO*^*Mac*^ model should be considered as model of partial *Lpcat3* deficiency in myeloid cells with significant residual *Lpcat3* activity. As a consequence, while the changes of phospholipid composition were broadly similar to those in fetal liver cell-derived macrophages with total *Lpcat3* deficiency (12) they were much less pronounced. Nevertheless, our data demonstrate that even a partial *Lpcat3* deficiency has a marked impact on the lipidomic profile of macrophages, thus underlying the major role of LPCAT3 in PL remodeling and PUFA metabolism in macrophages. While we confirm the reduction of EPA and AA in PCs, Pes, and pPEs as observed in *Lpcat3*^*−/−*^ macrophages, we extend these observations to PIs and PSs. However, LPCAT3 may not use LysoPI and LysoPS as direct substrates. Rather, our data suggest that the alterations in the FA composition of PCs and PEs induced by Lpcat3 deficiency secondarily affect other phospholipid subclasses. As previously observed, the reduced incorporation of arachidonate in PLs was associated with its redistribution toward other cellular lipids, such as cholesteryl esters. Increased C22:4 levels in different lipid subclasses (PEs, cholesteryl esters) were also observed suggesting a compensatory elongation of C20:4 to C22:4 as previously described (12). We speculate that the decrease in CE 20:5 relative abundance could be related to a competition between 20:4-CoA and 20:5-CoA as substrates for Acyl-CoA cholesterol acyl transferase (ACAT).

Although there is a controversy regarding the proinflammatory role of LPCAT3 in macrophages (4, 8, 12, 13), we could not find here any evidence for a proinflammatory macrophage phenotype associated with *Lpcat3* deficiency either in vitro or in vivo. While this could be due to the partial *Lpcat3* deficiency in our model, similar observations were made in fetal liver-derived macrophages with total *Lpcat3* deficiency (12). Moreover, in a similar model of myeloid *Lpcat3* deficiency, knockout of *Lpcat3* in macrophages has no effect on LXR repression of proinflammatory genes, such as *Cox2* or *Il1b* (14).

Transient *Lpcat3* inhibition has been associated with ER stress (8); however, we observed here that several markers of ER stress were significantly reduced in *Lpcat3KO*^*Mac*^ macrophages in basal conditions. Accordingly, Jiang *et al.* (13) also reported a decrease of the ER stress marker GRP78 (BIP) in *Lpcat3*-deficient macrophages.

Our group previously reported that *Lpcat3*^*−*/*−*^ macrophages displayed significant alteration of cholesterol homeostasis, including increased free-to-esterified cholesterol ratio and decreased cholesterol efflux (12). We could only partially reproduce this phenotype in the present study, where we observed only a significant reduction of cholesterol efflux while there were no changes in the expression of cholesterol transporters *Abca1*, *Abcg1*, or *ApoE*. We speculate that this is likely related to the residual LPCAT3 activity in *Lpcat3KO*^*Mac*^ cells. Indeed, it was shown that *Lpcat3*^*−*/*−*^ macrophages presented high levels of nonesterified 22:4 n-6 FA (adrenic acid), a potent LXR antagonist (21). Interestingly the level of this FA was not increased in *Lpcat3KO*^*Mac*^ cells. Nevertheless, our data suggest that *Lpcat3* deficiency may also affect cholesterol efflux by altering cell membrane lipid composition, a hypothesis that deserves future investigations. There was no increase in atherosclerotic lesions in *Ldlr*^*−/−*^ mice transplanted with *Lpcat3KO*^*Mac*^ bone marrow cells. Our data are in accordance with those of Jiang *et al.* with a similar mouse model (13). In contrast to these observations, transplantation of hematopoietic cells from mice with constitutive *Lpcat3* deficiency in *Ldlr*^*−/−*^ mice resulted in increased atherosclerotic lesions (12). However, these mice presented a total *Lpcat3* deficiency in all the hematopoietic lineages, including hematopoietic stem cells, which is likely to contribute to atherosclerosis development. Indeed, *Ldlr*^*−/−*^ mice transplanted with *Lpcat3*^*−/−*^ hematopoietic cells presented significantly higher monocyte counts with a relative increase in Ly-6C^high^ monocyte subsets, a phenotype that was not observed in the *Lpcat3KO*^*Mac*^ mouse model (12).

Obesity is another inflammatory chronic disease, in which macrophages play a critical role (22), and is a major risk factor for nonalcoholic fatty liver disease (NAFLD) (23). Here, we found that *Lpcat3KO*^*Mac*^ mice fed an HFD gained weight with a similar rate as control mice but developed liver metabolic alterations including hepatic steatosis. This was associated with increased liver gluconeogenesis and decreased FA oxidation, but without histological signs of liver inflammation. Transcriptomic profiling of liver myeloid cells did not reveal major differences between *Lpcat3KO*^*Mac*^ and control mice. This data suggests therefore that partial *Lpcat3* deficiency has no major impact on macrophage activation, as observed in vitro, despite changes in AA composition in liver myeloid cells. Accordingly, lipidomic profiling of the whole liver confirmed some alterations in AA homeostasis, including increased concentrations of AA-derived mediators such as TxB2 and LTB4, which are known to promote insulin resistance, inflammation (24, 25), antiplatelet activation (26). While no changes in inflammatory genes were observed, transcriptomic analysis by RNA sequencing shows alterations of cytochrome P450 pathways in the liver of *Lpcat3KO*^*Mac*^ mice fed an HFD with decrease of *Cyp4a12b* and increase of *Cyp4a14* expression. These genes are notably responsible for the formation of 20-HETE, which is involved in hepatic steatosis (27). Overexpression of *Cyp4a14* has been shown to be responsible for increase of FAT/CD36 expression that promotes liver triglyceride content (28). However, we did not find here any difference in 20-HETE liver content or CD36 expression.

While the molecular mechanisms linking hepatic steatosis and myeloid *Lpcat3* deficiency remain to be elucidated, our hypothesis is that changes in AA metabolism-restricted liver myeloid cells may secondarily impact AA homeostasis in the whole liver leading to metabolic disorders and TG accumulation. In conclusion, the role of LPCAT3 in controlling macrophage functions seems to be complex. It will deserve future studies with new experimental approaches and models.

## Conflict of interest

The authors declare that they have no conflicts of interest with the contents of this article.
