# Supplementary material for: Deletion of lysophosphatidylcholine acyltransferase 3 in myeloid cells worsens hepatic steatosis after a high-fat diet
Source: J Lipid Res. 2020 Dec 17;62:100013. doi: 10.1194/jlr.RA120000737 (PMC7859853; doi:10.1194/jlr.RA120000737)
Supplement: Supplemental Figures S1 to S3 [file mmc1.pdf]

Molecular species	logfold	fold	WT1	WT2	WT3	WT4	Lpcat3 KOMAC1	Lpcat3 KOMAC2	Lpcat3 KOMAC3	Lpcat3 KOMAC4
**34:2 PI**	-0,24504	0,84379	0,27922	0,18497	0,30126	0,20446	0,18911	0,21163	0,22904	0,18862
**34:1 PI**	-0,24073	0,84632	0,72690	0,49306	0,89094	0,60144	0,66134	0,50544	0,57604	0,55269
**34:0 PI**	-0,25926	0,83552	0,11902	0,13681	0,21822	0,24934	0,10866	0,16752	0,20824	0,12000
**36:4 PI**	0,05334	1,03766	2,43237	2,26254	1,87350	1,95770	2,49605	1,96674	1,87877	2,50566
**36:3 PI**	-0,03712	0,97460	0,61595	0,49557	0,51369	0,53412	0,52133	0,49884	0,55585	0,52845
**36:2 PI**	-0,26343	0,83311	3,75220	2,89442	4,41248	3,20365	3,01667	2,76129	3,20685	2,89760
**36:1 PI**	-0,20853	0,86542	1,39687	1,17933	1,98602	1,57498	1,39458	1,38545	1,55387	0,97736
**36:0 PI**	0,07071	1,05023	0,04014	0,03943	0,03069	0,05050	0,02767	0,04015	0,03113	0,06989
**38:5 PI**	0,27518	1,21015	6,95166	6,20464	6,75987	6,32193	8,21650	7,65013	7,72587	8,15945
**38:4 PI**	0,02512	1,01756	68,65970	71,47677	67,69715	70,70192	71,72045	70,25511	69,69983	71,75163
**40:7 PI**	0,26433	1,20108	0,19495	0,19294	0,25657	0,28691	0,32307	0,29523	0,30593	0,19442
**40:6 PI**	-0,27132	0,82856	1,78002	1,49211	1,54078	1,56269	1,38477	1,29084	1,29094	1,31601
**40:5 PI**	-0,32037	0,80086	10,74637	10,74877	11,33414	10,50482	6,71556	10,10742	9,78308	8,09870
**40:4 PI**	0,38706	1,30773	2,30461	2,19862	2,18468	2,24554	3,22426	2,86422	2,95455	2,63949
**C12:0**	-0,28841	0,81881	1,57279	1,35149	1,17403	1,27944	1,06408	0,60783	1,46300	1,26843
**C14:0**	0,02467	1,01725	1,75980	1,78789	1,55087	1,75647	1,93017	1,71772	1,64197	1,68340
**C15:0**	-0,20766	0,86594	0,45815	0,51157	0,39809	0,53341	0,50651	0,39631	0,38941	0,35412
**C16:1 n-9**	-0,26738	0,83083	0,48593	1,06577	0,36583	0,47335	0,59632	0,45899	0,42684	0,50427
**C16:1 n-7**	0,46131	1,37679	0,23282	0,20046	0,20292	0,17632	0,24731	0,20272	0,15614	0,51250
**C16:0**	-0,01242	0,99143	48,22471	47,75893	48,27577	49,74666	45,84559	49,26135	49,59007	47,64553
**C17:0**	0,08666	1,06191	0,44342	0,40816	0,39294	0,43409	0,52022	0,42912	0,40573	0,42747
**C18:1 n-9**	0,05084	1,03587	2,44874	2,93555	2,60368	2,23240	2,85830	2,47858	2,01891	3,23117
**C18:1 n-7**	0,08894	1,06359	1,11803	1,05966	1,25186	1,00256	1,31443	1,21721	0,90066	1,28164
**C18:0**	-0,01938	0,98666	39,24501	37,24411	37,47420	37,29541	37,99164	37,28071	38,32450	35,64347
**C18:2 n-6**	-0,07153	0,95163	0,95053	1,15073	0,78276	0,64995	0,87007	0,79057	0,77913	0,92324
**C18:3 n-6**	0,86100	1,81629	0,01023	0,01191	0,00824	0,00816	0,01242	0,00913	0,00930	0,03917
**C18:3 n-3**	-0,54769	0,68411	0,00913	0,01470	0,00568	0,00379	0,00596	0,00515	0,00496	0,00671
**C19:0**	0,05468	1,03863	0,06321	0,06104	0,05911	0,06661	0,07504	0,06103	0,06277	0,06079
**C20:0**	-0,13327	0,91176	0,43452	0,37378	0,37233	0,38000	0,36625	0,34813	0,38094	0,32759
**C20:1 n-9**	0,17436	1,12847	0,05276	0,09677	0,14677	0,11631	0,12440	0,11715	0,10911	0,11495
**C20:3 n-6**	0,30415	1,23469	0,07568	0,10652	0,14822	0,07779	0,12405	0,10331	0,06957	0,20708
**C20:3 n-9**	0,56503	1,47942	0,06494	0,09654	0,17615	0,08717	0,14718	0,12742	0,06973	0,28413
**C20:4 n-6**	0,48904	1,40352	0,73522	1,19726	1,39533	1,17490	1,74235	1,52149	1,02752	2,02827
**C20:5 n-3**	1,22408	2,33606	0,05824	0,08117	0,12818	0,06902	0,08428	0,10331	0,04170	0,55707
**C21:0**	-0,14672	0,90330	0,01397	0,01417	0,01076	0,01550	0,01266	0,01087	0,01236	0,01325
**C22:0**	0,13383	1,09721	0,02987	0,03009	0,02793	0,03230	0,03745	0,02793	0,02582	0,04066
**C22:1 n-9**	-0,42261	0,74608	0,01641	0,02027	0,06630	0,04820	0,02536	0,01935	0,02441	0,04367
**C22:1 n-7**	0,42347	1,34115	0,00382	0,00447	0,01041	0,00607	0,00953	0,00762	0,00543	0,01063
**C22:4 n-6**	0,24633	1,18619	0,41757	0,70368	1,01404	0,83354	1,30452	0,86006	0,79846	0,55855
**C22:5 n-6**	0,25020	1,18937	0,01838	0,03962	0,02861	0,04551	0,03938	0,04470	0,03662	0,03645
**C22:5 n-3**	0,26433	1,20108	0,43531	0,71918	0,87352	0,57595	0,92452	0,81126	0,53380	0,85799
**C22:6 n-3**	0,33781	1,26384	0,42395	0,69316	0,81159	0,53913	0,86266	0,72367	0,47185	1,06077
**C24:5**	0,11780	1,08508	0,00677	0,01097	0,01105	0,01040	0,01215	0,00991	0,00910	0,01135
**C24:6 n-3**	-0,06139	0,95834	0,00923	0,01262	0,01544	0,00893	0,01002	0,01043	0,00796	0,01587
**C24:4**	0,68885	1,61200	0,00650	0,01317	0,01233	0,01690	0,02382	0,02516	0,02328	0,00656
**C23:0**	-0,09314	0,93748	0,01294	0,01242	0,00921	0,01609	0,01575	0,01098	0,01032	0,01044
**C24:1 n-9**	0,36907	1,29152	0,03856	0,05738	0,08673	0,05891	0,08732	0,08444	0,04253	0,09771
**C24:0**	0,10848	1,07809	0,05423	0,07045	0,05545	0,09632	0,09452	0,06058	0,05665	0,08629
**C25:0**	-0,24549	0,84353	0,02375	0,02745	0,01894	0,04391	0,03693	0,01751	0,02437	0,01739
**C26:0**	-0,23152	0,85174	0,04489	0,05688	0,03475	0,08850	0,07685	0,03830	0,04508	0,03142
**C14:0 CE**	-0,55927	0,67864	0,65550	0,66707	0,59550	0,35816	0,36621	0,32610	0,35335	0,49909
**C16:1 CE**	-0,29131	0,81716	3,88443	3,97809	2,73370	2,02970	2,47832	2,15075	2,20816	3,48014
**C16:0 CE**	-0,25859	0,83590	3,32557	2,96795	3,14615	2,19730	2,33977	1,99854	2,34653	3,04255
**C18:3 CE**	0,18416	1,13616	0,49344	0,50724	0,37089	0,24161	0,43910	0,30375	0,42668	0,66331
**C18:2 CE**	0,01669	1,01164	6,02667	5,80871	4,69420	3,56650	5,05438	4,94318	4,59797	5,73437
**C18:1 CE**	-0,10688	0,92859	21,29528	19,35511	20,48031	16,15049	17,30535	16,76289	18,23934	19,45502
**C18:0 CE**	-0,11130	0,92575	1,45984	1,34654	1,57831	1,26672	1,15858	1,14944	1,35153	1,57223
**C20:5 CE**	-0,37698	0,77005	3,64854	3,80273	3,54246	4,20476	3,08785	2,73150	2,91797	2,96623
**C20:4 CE**	0,71264	1,63880	10,60634	12,58102	10,64401	13,50115	19,48510	20,70020	19,15327	18,22990
**C20:3 CE**	-0,38862	0,76386	7,38954	6,92693	9,67137	9,76459	6,32488	7,10594	6,66173	5,68951
**C20:2 CE**	-0,27938	0,82394	3,16595	2,89061	3,36832	3,31949	2,59697	2,96132	2,69557	2,24678
**C20:1 CE**	-0,13010	0,91377	1,30253	1,16515	1,62208	1,63820	1,16918	1,35099	1,41157	1,30228
**C20:0 CE**	-0,12380	0,91777	0,06387	0,09454	0,10282	0,10798	0,07775	0,10400	0,06142	0,09568
**C22:6 CE**	-0,31534	0,80366	8,82152	8,97747	7,73142	7,71205	7,00833	6,67683	6,63965	6,39089
**C22:5 CE**	-0,41125	0,75197	15,39467	16,08215	16,56086	17,59238	13,32163	11,86368	12,47203	11,69461
**C22:4 CE**	0,66067	1,58082	6,27408	7,19929	6,67301	8,63050	11,24429	11,98875	11,75764	10,50030
**C22:3 CE**	-0,26873	0,83005	1,06216	1,10939	1,47144	1,55259	1,07377	1,20736	1,11279	0,91867
**C22:2 CE**	0,09685	1,06943	0,34007	0,33505	0,33608	0,36259	0,32878	0,45089	0,36925	0,32026
**C22:1 CE**	-0,05386	0,96336	0,36507	0,31331	0,42197	0,44864	0,32160	0,39753	0,40517	0,36794
**C22:0 CE**	0,43279	1,34984	0,11404	0,00618	0,09002	0,13726	0,10547	0,12513	0,11516	0,12331
**C24:6 CE**	-0,35863	0,77990	0,74301	0,75939	0,71771	0,66098	0,57943	0,54956	0,57699	0,54099
**C24:5 CE**	-0,08669	0,94168	1,15086	1,11240	0,89415	0,95362	1,07753	0,89013	0,94323	0,96037
**C24:4 CE**	1,15652	2,22919	0,59648	0,04628	0,51072	0,60739	0,99418	1,00255	0,92458	1,00401
**C24:3 CE**	0,12381	1,08961	0,23394	0,21159	0,19753	0,25519	0,22665	0,22166	0,26392	0,26651
**C24:2 CE**	0,22224	1,16655	0,34327	0,41651	0,33477	0,50470	0,43839	0,51581	0,43067	0,48073
**C24:1 CE**	-0,08870	0,94037	1,01966	1,14377	1,34249	1,97315	1,20473	1,35691	1,35083	1,23987
**C24:0 CE**	-0,11569	0,92294	0,22368	0,19553	0,16772	0,26230	0,19176	0,16459	0,21299	0,21446
**16:0/18:2 PS**	-0,04568	0,96884	0,72658	0,42890	0,60065	0,50499	0,56430	0,46383	0,50062	0,66189
**16:0/18:1 PS**	-0,03531	0,97582	5,58573	4,83326	6,19444	5,42095	5,28900	5,47073	5,36496	5,37697
**17:0/17:0 PS**	0,06679	1,04739	9,63713	8,57160	7,22972	8,12336	9,76727	7,02351	7,78067	10,58069
**18:1/18:2 PS**	-0,14669	0,90332	0,63088	0,48827	0,52030	0,47331	0,53265	0,31587	0,40753	0,65245
**18:0/18:2 PS**	-0,24402	0,84439	12,19012	11,45213	10,96394	11,11253	8,59101	10,82515	10,17902	9,00912
**18:0/18:1 PS**	0,07751	1,05520	27,23460	29,21683	37,83598	35,30063	35,16424	35,47117	34,35942	31,74587
**18:1/20:4 PS**	-0,68646	0,62138	0,60247	0,48330	0,64678	0,56378	0,39214	0,28009	0,30724	0,44742
**18:0/20:4 PS**	-0,50388	0,70521	10,79684	10,75709	9,42936	10,90992	6,75248	7,89629	7,67006	7,22463
**18:0/20:3 PS**	-0,29887	0,81289	4,73768	5,88887	6,12692	5,86514	4,73667	4,84397	4,37621	4,42960
**18:1/22:6 PS**	0,68599	1,60881	0,34093	0,21907	0,19813	0,15730	0,40500	0,24641	0,30782	0,51353
**18:0/22:6 PS**	0,14445	1,10531	15,91651	16,74822	11,79660	12,86414	14,48119	16,36826	15,62917	16,88389
**18:0/22:5 PS**	0,32534	1,25296	11,60053	10,91246	8,45718	8,70395	13,32403	10,79472	13,11729	12,47394
**p16:0/16:0 pPE**	0,00679	1,00472	0,07115	0,09856	0,07469	0,09692	0,08623	0,08258	0,09414	0,07997
**p18:0/14:0 pPE**	-0,27447	0,82675	0,02571	0,03077	0,02665	0,02757	0,02356	0,02278	0,02088	0,02431
**32:2 PE**	0,67438	1,59591	0,04137	0,03812	0,04456	0,03373	0,05936	0,07759	0,05723	0,05762
**32:1 PE**	0,42519	1,34275	0,98767	0,89338	1,05564	0,85356	1,23728	1,44535	1,23926	1,16748
**32:0 PE**	0,05866	1,04150	0,24536	0,29860	0,26478	0,27669	0,31282	0,24813	0,28194	0,28758
**p16:0/18:2 pPE**	-0,01861	0,98718	0,33237	0,29966	0,40231	0,39286	0,31913	0,42162	0,35853	0,30965
**p16:0/18:1 pPE**	-0,12505	0,91697	1,74098	1,57956	2,68639	2,49131	1,72400	2,47518	2,09350	1,49998
**p18:0/16:0 pPE**	-0,35774	0,78039	0,04723	0,06538	0,04410	0,06364	0,04928	0,03527	0,04177	0,04563
**p18:1/17:0 pPE**	0,19668	1,14606	0,01635	0,01288	0,01607	0,01344	0,01935	0,01795	0,01412	0,01590
**34:2 PE**	0,36056	1,28393	1,21522	1,06592	1,33283	1,05610	1,43151	1,66980	1,51652	1,37820
**34:1 PE**	0,25109	1,19011	4,63866	4,21755	4,75339	4,35542	5,34152	5,41979	5,28602	5,33299
**p16:0/20:5 pPE**	-1,67422	0,31334	0,48363	0,38126	0,69042	0,57203	0,15829	0,21704	0,17127	0,11997
**p16:0/20:4 pPE**	-0,57982	0,66905	8,23188	8,59055	8,83924	9,94969	6,13527	6,31647	5,86913	5,50476
**p18:1/18:3 pPE**	0,42657	1,34403	0,01678	0,01513	0,01759	0,01816	0,02239	0,02529	0,02060	0,02266

Molecular species	logfold	fold	WT1	WT2	WT3	WT4	Lpcat3 KOMAC1	Lpcat3 KOMAC2	Lpcat3 KOMAC3	Lpcat3 KOMAC4
**p16:0/20:3 pPE**	-0,61030	0,65506	0,48828	0,43331	0,59663	0,56792	0,38743	0,39071	0,33986	0,24855
**p18:1/18:2 pPE**	0,08414	1,06005	0,11125	0,10199	0,13021	0,11655	0,11913	0,13317	0,12674	0,10860
**p18:1/18:1 pPE**	-0,08713	0,94140	0,57563	0,54031	0,76663	0,74584	0,59436	0,71952	0,63846	0,52202
**p18:0/18:2 pPE**	-0,18386	0,88035	0,15288	0,14996	0,18777	0,22059	0,13382	0,18316	0,16153	0,14759
**p18:1/18:0 pPE**	-0,32334	0,79922	0,01755	0,01831	0,01441	0,01523	0,01423	0,01240	0,01495	0,01076
**p18:0/18:1 pPE**	-0,29675	0,81409	0,59225	0,60839	0,86169	0,84558	0,51954	0,69094	0,65073	0,50608
**35:2 PE**	0,33668	1,26285	0,26800	0,22604	0,22255	0,17592	0,29543	0,28359	0,25704	0,29103
**p16:0/20:0 pPE**	-0,80834	0,57104	0,00727	0,00712	0,01139	0,01407	0,00546	0,00757	0,00731	0,00243
**p18:0/18:0 pPE**	-0,36918	0,77422	0,02476	0,01952	0,02703	0,03071	0,01692	0,01955	0,02268	0,01984
**35:1 PE**	0,19448	1,14431	0,73018	0,69081	0,67653	0,63207	0,75083	0,73490	0,78814	0,84962
**36:4 PE**	-0,07255	0,95096	2,25679	2,26267	2,14248	2,04078	2,15793	2,25132	1,88247	1,98418
**36:3 PE**	0,09692	1,06949	0,99004	0,86764	0,90130	0,80592	0,89166	1,01993	0,95229	0,94873
**36:2 PE**	0,22116	1,16567	5,75588	5,24375	6,00842	5,18123	6,19237	6,67793	6,57334	6,42184
**36:1 PE**	0,10837	1,07801	9,19191	8,93146	9,52970	9,49805	9,46728	9,70159	10,68593	10,19461
**p16:0/22:6 pPE**	-0,22295	0,85681	3,17803	3,04286	3,27508	3,05951	2,78195	2,74883	2,70558	2,52130
**p18:1/20:5 pPE**	-1,56795	0,33729	0,16908	0,15048	0,28015	0,22440	0,06630	0,09510	0,07135	0,04521
**p16:0/22:5 pPE**	0,00601	1,00418	2,87433	2,70016	2,68581	2,42101	2,80344	2,61699	2,66519	2,64032
**p18:1/20:4 pPE**	-0,53249	0,69136	3,32437	3,40865	3,77687	4,15826	2,65175	2,62915	2,44981	2,41026
**p18:0/20:5 pPE**	-1,85930	0,27561	0,19431	0,17921	0,28207	0,23559	0,06472	0,07776	0,05811	0,04502
**p16:0/22:4 pPE**	0,81442	1,75859	1,23177	1,29466	1,08442	1,12952	2,16609	1,99266	2,04545	2,13214
**p18:1/20:3 pPE**	0,54476	1,45878	0,71643	0,79179	0,74751	0,74897	0,96429	1,08478	1,16436	1,16976
**p18:0/20:4 pPE**	-0,81108	0,56996	3,82918	4,26820	3,97565	4,70928	2,48564	2,34602	2,42121	2,31228
**p16:0/22:3 pPE**	-0,07934	0,94649	0,19533	0,24138	0,23227	0,24700	0,19759	0,22623	0,24159	0,20155
**p18:1/20:2 pPE**	-0,26859	0,83013	0,05812	0,05264	0,07871	0,07059	0,04682	0,06862	0,05244	0,04801
**p18:0/20:3 pPE**	-0,46369	0,72513	0,36558	0,38100	0,45425	0,48612	0,28747	0,34156	0,31750	0,27671
**p16:0/22:2 pPE**	-0,30452	0,80971	0,03037	0,03555	0,04343	0,04191	0,02511	0,03330	0,03876	0,02531
**p18:1/20:1 pPE**	-0,47397	0,71998	0,05221	0,05008	0,08680	0,08497	0,03919	0,06690	0,05332	0,03792
**p18:0/20:2 pPE**	-0,43106	0,74172	0,04917	0,04909	0,07068	0,07699	0,04377	0,04747	0,05478	0,03639
**p16:0/22:1 pPE**	-0,72695	0,60418	0,03609	0,04336	0,05687	0,06096	0,02739	0,03663	0,03533	0,01985
**p18:1/20:0 pPE**	-0,54664	0,68461	0,00650	0,01068	0,01312	0,01137	0,00767	0,00869	0,00654	0,00563
**p18:0/20:1 pPE**	-0,66210	0,63196	0,04718	0,04933	0,08310	0,08687	0,03582	0,05427	0,05273	0,02559
**38:6 PE**	0,12723	1,09220	1,79623	1,72729	1,66775	1,35350	1,84562	1,89141	1,67407	1,73705
**38:5 PE**	-0,04218	0,97118	3,71989	3,67964	3,80647	3,58223	3,65837	3,81790	3,48680	3,39901
**38:4 PE**	-0,18368	0,88045	13,58354	14,38228	12,74229	14,13510	12,61210	11,49686	11,72765	12,45025
**38:3 PE**	-0,30117	0,81159	1,82247	1,77410	1,59772	1,60427	1,27865	1,55892	1,32214	1,35795
**38:2 PE**	-0,20365	0,86835	0,65270	0,64585	0,85738	0,85021	0,62159	0,67460	0,72633	0,58787
**38:1 PE**	-0,30857	0,80744	0,18497	0,20185	0,20152	0,21478	0,13922	0,17571	0,18124	0,15230
**p18:1/22:5 pPE**	0,06254	1,04430	1,07607	1,00583	0,97549	0,89164	1,09392	0,98578	0,99789	1,04638
**p18:0/22:6 pPE**	-0,44622	0,73396	1,30535	1,36149	1,29981	1,29466	1,01360	0,90167	0,98574	0,96061
**p18:1/22:4 pPE**	1,00080	2,00111	0,50593	0,59411	0,44911	0,44467	0,97919	0,87475	1,01300	1,12291
**p18:0/22:5 pPE**	-0,21892	0,85921	1,34528	1,48015	1,25366	1,24084	1,16165	1,04723	1,15404	1,20802
**p16:0/24:4 pPE**	0,30684	1,23699	0,09529	0,09151	0,07375	0,07455	0,09815	0,09176	0,10578	0,11884
**p18:1/22:3 pPE**	0,62088	1,53781	0,20127	0,24201	0,19194	0,19196	0,27569	0,28560	0,35361	0,35714
**p18:0/22:4 pPE**	0,65255	1,57194	0,48219	0,53414	0,37149	0,41362	0,70071	0,60118	0,71432	0,81556
**p16:0/24:3 pPE**	-0,11222	0,92516	0,00858	0,01637	0,01191	0,02091	0,01049	0,01242	0,01366	0,01687
**p18:1/22:2 pPE**	-0,71304	0,61003	0,01504	0,01245	0,01679	0,01689	0,01016	0,01247	0,00845	0,00624
**p18:0/22:3 pPE**	-0,01066	0,99264	0,07747	0,09152	0,06680	0,08224	0,07195	0,08262	0,07928	0,08184
**p16:0/24:2 pPE**	-0,53613	0,68962	0,00209	0,00291	0,00444	0,00464	0,00341	0,00267	0,00241	0,00122
**p18:1/22:1 pPE**	0,01534	1,01069	0,01002	0,00960	0,01489	0,01131	0,01242	0,01359	0,01389	0,00641
**p18:0/22:2 pPE**	-0,19349	0,87449	0,00491	0,00796	0,00994	0,00957	0,00654	0,00852	0,00793	0,00533
**p18:0/22:1 pPE**	-0,68030	0,62403	0,00758	0,01162	0,01209	0,01757	0,00794	0,00745	0,01087	0,00423
**40:7 PE**	0,26978	1,20562	1,65449	1,49414	1,63115	1,30944	1,82842	1,98382	1,78679	1,74228
**40:6 PE**	0,11392	1,08217	5,97912	5,88583	5,23760	5,18508	6,13413	5,82435	5,88812	6,27236
**40:5 PE**	0,37052	1,29282	6,14222	6,20007	4,91138	4,87828	7,25073	6,64665	7,12513	7,59005
**40:4 PE**	0,90159	1,86812	2,62344	2,93569	2,11795	2,21789	4,59469	4,01082	4,55787	5,32162
**40:3 PE**	0,25818	1,19597	0,21428	0,24187	0,20076	0,22332	0,25859	0,24252	0,27260	0,27900
**40:2 PE**	-0,00917	0,99366	0,04692	0,05507	0,05401	0,07327	0,05786	0,05030	0,06465	0,05500
**40:1 PE**	-0,48880	0,71262	0,02620	0,02822	0,03832	0,03837	0,02165	0,02126	0,03543	0,01510
**42:6 PE**	0,32766	1,25498	0,28705	0,28460	0,25269	0,26966	0,34764	0,32297	0,34373	0,35860
**42:5 PE**	0,69315	1,61681	0,29273	0,28530	0,19903	0,20805	0,41261	0,32845	0,39438	0,45731
**42:4 PE**	0,71071	1,63661	0,18630	0,23699	0,14741	0,15630	0,29952	0,21234	0,30646	0,37149
**42:3 PE**	0,08829	1,06311	0,01973	0,02460	0,01721	0,02539	0,01343	0,02335	0,02722	0,02842
**42:2 PE**	0,10158	1,07295	0,00848	0,00891	0,00829	0,00889	0,01031	0,00837	0,00893	0,00949
**42:1 PE**	0,08355	1,05962	0,00709	0,00834	0,00676	0,00600	0,00768	0,00561	0,00913	0,00744
**28:0 PC**	0,42522	1,34278	0,29924	0,30009	0,23814	0,18841	0,33002	0,35585	0,30893	0,38274
**30:0 PC**	0,11611	1,08381	8,19260	8,99666	7,88520	6,87940	8,35665	9,06936	8,83850	8,36727
**32:2 PC**	0,38265	1,30374	0,50936	0,47016	0,52476	0,41082	0,60583	0,62756	0,61861	0,64478
**32:1 PC**	0,20035	1,14898	10,54626	10,47889	10,92113	9,28556	11,56218	12,42728	12,23617	11,14872
**32:0 PC**	0,12442	1,09007	10,47387	11,32195	10,40925	12,59691	12,91203	10,94969	11,78726	13,18826
**34:2 pPC**	-0,34005	0,79001	0,04155	0,03459	0,04309	0,04725	0,03202	0,02783	0,02971	0,04195
**33:2 PC**	0,14721	1,10742	0,45265	0,43219	0,39597	0,31287	0,41933	0,46569	0,47484	0,40500
**34:1 pPC**	-0,04298	0,97065	0,58410	0,54817	0,58096	0,54594	0,54067	0,51515	0,55163	0,58540
**33:1 PC**	0,30832	1,23827	1,75414	1,65438	1,53400	1,32782	1,92169	1,82573	1,94545	2,07149
**34:0 pPC**	-0,01285	0,99114	3,60576	3,34777	3,71570	3,55393	3,70941	3,33373	3,58575	3,46818
**34:4 PC**	0,08946	1,06397	0,22894	0,28006	0,19389	0,21146	0,24744	0,23231	0,22685	0,26624
**34:3 PC**	0,02010	1,01403	0,82376	0,87556	0,86406	0,68566	0,78839	0,87401	0,87063	0,76160
**34:2 PC**	-0,03159	0,97834	5,49606	5,12854	5,83066	4,87772	5,05056	5,34381	5,42068	5,05593
**34:1 PC**	0,05535	1,03911	18,85176	17,19967	18,81499	18,29195	19,02005	18,41150	18,94870	19,63927
**36:4 pPC**	-1,35848	0,38999	0,13355	0,11390	0,17258	0,17458	0,05702	0,06338	0,06471	0,04679
**35:4 PC**	-0,24540	0,84358	0,20855	0,28345	0,17562	0,20195	0,17779	0,20041	0,18077	0,17459
**36:3 pPC**	-0,97367	0,50921	2,23893	2,47429	2,41539	3,12697	1,39142	1,24592	1,30469	1,28020
**35:2 PC**	0,03874	1,02721	0,59717	0,48264	0,48729	0,42840	0,50444	0,49524	0,50732	0,54280
**36:1 pPC**	0,06823	1,04843	0,46676	0,42985	0,61093	0,53643	0,53674	0,60744	0,52344	0,47535
**35:1 PC**	-0,20328	0,86857	0,93639	0,94031	0,70975	0,86284	0,76789	0,73954	0,70950	0,77903
**36:0 pPC**	-0,17805	0,88390	0,14766	0,13106	0,17492	0,15816	0,13762	0,15502	0,15132	0,09681
**36:5 PC**	-0,46542	0,72426	1,36395	1,52956	1,47546	1,50702	1,05072	1,19860	1,10831	0,89811
**36:4 PC**	-0,25668	0,83701	4,75907	5,18341	4,62706	5,56569	4,27536	4,31564	4,13462	4,12781
**36:3 PC**	-0,30573	0,80903	2,46761	2,18747	2,49922	2,17540	1,71273	2,18951	1,87571	1,77007
**36:2 PC**	-0,09791	0,93439	7,06046	6,66798	7,58103	6,88170	6,44787	6,79999	6,73493	6,35871
**36:1 PC**	-0,00299	0,99793	4,10754	4,18849	4,47392	4,51270	4,13381	4,65544	4,44153	4,01615
**38:6 PC**	0,15510	1,11350	1,49515	1,13664	1,37852	1,29153	1,53803	1,58486	1,27171	1,50900
**38:5 PC**	-0,15298	0,89939	3,75681	4,10866	3,55553	4,20415	3,57123	3,51208	3,47321	3,49658
**38:4 PC**	-0,57144	0,67294	3,18290	3,79583	3,03358	4,04397	2,44130	2,31220	2,25362	2,45196
**38:3 PC**	-0,52313	0,69586	0,52713	0,47978	0,46458	0,60759	0,39012	0,43957	0,31144	0,30561
**38:2 PC**	-0,22210	0,85732	0,27284	0,33311	0,36295	0,24258	0,18200	0,34815	0,27645	0,23203
**38:1 PC**	-0,01325	0,99086	0,14531	0,13262	0,14963	0,16277	0,14858	0,16508	0,15736	0,11392
**38:0 PC**	0,04879	1,03439	1,36918	1,32361	1,13592	1,30737	1,39198	0,97849	1,22060	1,72167
**40:8 PC**	-0,20758	0,86599	0,20019	0,24444	0,20068	0,24805	0,19071	0,21540	0,19714	0,17039
**40:7 PC**	0,25264	1,19139	0,53210	0,51358	0,48331	0,50791	0,61482	0,61664	0,61181	0,58346
**40:6 PC**	0,33315	1,25976	0,63062	0,50734	0,54607	0,48890	0,70161	0,66482	0,67098	0,69996
**40:5 PC**	0,30055	1,23161	1,12691	1,22272	0,94649	1,09015	1,40237	1,32193	1,28732	1,39057
**40:4 PC**	0,69101	1,61441	0,41318	0,52060	0,38777	0,45749	0,73756	0,71513	0,68784	0,73159

Supplemental Figure 1 : Detailed phospholipid subclasses concentration of *Lpcat3* deficient macrophages. Phosphatidylcholine (PC), phosphatidylethanolamine (PE) and associated plasmalogens (pPE and pPC), phosphatidylinositol (PI), phosphatidylserine (PS) and cholesterol ester (CE) and free fatty acid composition of macrophages deficient for *Lpcat3* gene (n=4 in each group).

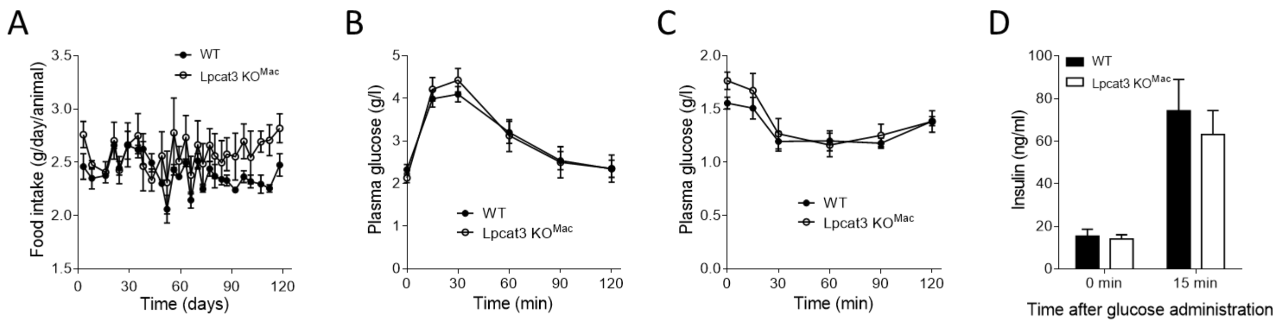

Supplemental Figure 2 : Metabolic parameters in *Lpcat3KO*^{Mac} mice fed a HFD for 16 Weeks (A) Food intake during 16 weeks of HFD. (B and C) Oral glucose tolerance test (B) and insulin tolerance test (C) of 6 hours fasting WT and *Lpcat3KO*^{Mac} mice (D) Insulin secretion after oral glucose administration. (n=7vs13) Data are expressed as mean +/- SEM.

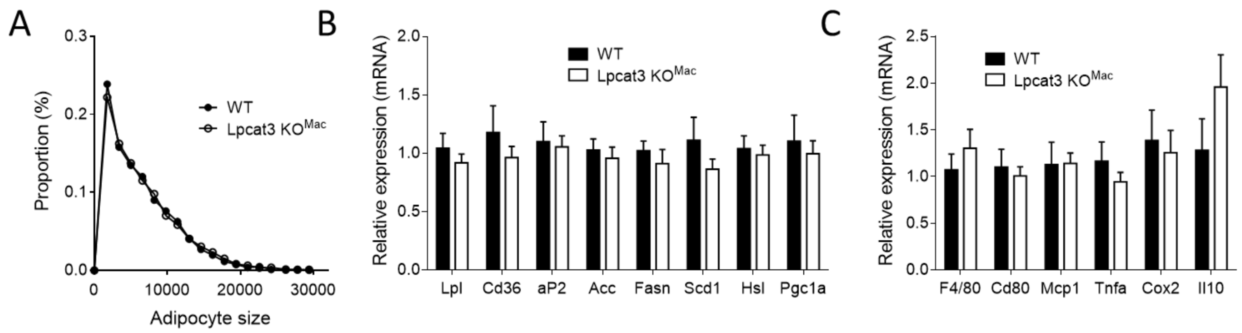

Supplemental Figure 3 : Analysis of adipose tissue from *Lpcat3KO^{Mac}* mice after 16 weeks of HFD. (A) Adipocyte size using adipose tissue histological sections. (B and C) mRNA levels of genes involved in adipocyte metabolism (B) and adipose tissue inflammation (C). n=7vs13, Data are expressed as mean + SEM.
